# Phospholamban p.Leu39* Cardiomyopathy Compared with Other Sarcomeric Cardiomyopathies: Age-Matched Patient Cohorts and Literature Review

**DOI:** 10.3390/jcdd11020041

**Published:** 2024-01-28

**Authors:** Andreea Sorina Afana, Laura Vasiliu, Radu Sascău, Robert Daniel Adam, Cristina Rădulescu, Sebastian Onciul, Eliza Cinteză, Adela Chirita-Emandi, Ruxandra Jurcuț

**Affiliations:** 1 Expert Center for Genetic Cardiovascular Diseases, Emergency Institute for Cardiovascular Diseases, 258 Fundeni Street, 022328 Bucharest, Romania; andreea.afana@gmail.com (A.S.A.);; 2 Emergency Clinical County Hospital Craiova, 1 Tabaci Street, 200642 Craiova, Romania; 3 Cardiology Department, University of Medicine and Pharmacy Craiova, 2 Petru Rares Street, 200349 Craiova, Romania; 4 Institute of Cardiovascular Diseases “Prof. Dr. George I.M. Georgescu”, 700503 Iași, Romania; laura.tapoi@yahoo.com (L.V.); radu.sascau@gmail.com (R.S.); 5 Cardiology Department, University of Medicine and Pharmacy “Grigore T. Popa”, 700115 Iași, Romania; 6 Cardiology Department, University of Medicine and Pharmacy “Carol Davila”, 8 Eroii Sanitari Blvd., 050474 Bucharest, Romania; cristina.radulescu@umfcd.ro (C.R.); sebastian.onciul@umfcd.ro (S.O.); eliza.cinteza@umfcd.ro (E.C.); 7 Emerald Medical Center, 75 Nicolae G. Caramfil Street, 077190 Bucharest, Romania; 8 Emergency Clinical Hospital Floreasca, 8 Calea Floreasca, 014461 Bucharest, Romania; 9 Department of Pediatric Cardiology, “Marie Curie” Emergency Children’s Hospital, 41451 Bucharest, Romania; 10 Department of Microscopic Morphology, Genetics Discipline, Center of Genomic Medicine, University of Medicine and Pharmacy “Victor Babeș” Timișoara, 2 Piaţa Eftimie Murgu Street, 300041 Timişoara, Romania; adela.chirita@umft.ro; 11 Regional Center of Medical Genetics Timiș, Clinical Emergency Hospital for Children “Louis Țurcanu” Timișoara, 2 Doctor Iosif Nemoianu Street, 300011 Timișoara, Romania

**Keywords:** phospholamban, hypertrophic cardiomyopathy, genetic testing

## Abstract

Hypertrophic cardiomyopathy (HCM) is a heterogeneous genetic disorder, most often caused by sarcomeric gene mutations, with a small proportion due to variants in non-sarcomeric loci. Phospholamban (PLN) is a phosphoprotein associated with the cardiac sarcoplasmic reticulum, a major determinant of cardiac contractility and relaxation. We conducted a retrospective study to determine the prevalence, phenotypical spectrum and clinical course of patients carrying the *PLN* p.Leu39* variant. A cohort including 11 *PLN* patients was identified among all patients with HCM (9/189, 4.8%) and DCM (2/62, 3.2%) who underwent genetic testing from two tertiary centers and five more were detected through cascade screening. Complete phenotyping was performed. *PLN* p.Leu39* variant-driven cardiomyopathy presented mostly as hypertrophic, with frequent progression to end-stage dilated HCM. We proceeded to compare these results to a similar analysis of a control cohort consisting of age-matched individuals that inherited pathogenic or likely pathogenic variants in common sarcomeric genes (*MYBPC3/MYH7*). Overall, the clinical characteristics and examination findings of patients carrying *PLN* p.Leu39* were not different from patients with cardiomyopathy related to sarcomeric mutations except for the presence of pathological Q waves and the incidence of non-sustained ventricular arrhythmias, which were higher in *PLN* patients than in those with *MYBPC3/MYH7*-related diseases.

## 1. Introduction

Hereditary cardiomyopathies are a heterogeneous group of primary myocardial diseases caused by genetic variants in which the heart muscle is structurally and functionally abnormal, resulting in various phenotypes. Hypertrophic cardiomyopathy is the most common subtype, representing a phenotype of left ventricular hypertrophy unexplained entirely by abnormal loading conditions. It is one of the leading causes of sudden cardiac death (SCD) in young people and athletes [1] and one of the most common inherited cardiovascular diseases, affecting 1 in 500 individuals. It is mostly caused by disease-causing variants in genes encoding sarcomeric proteins, which are identified in up to 60% of cases of the disease. Dilated cardiomyopathy (DCM) is also an important genetic heart disease defined by the presence of left ventricular dilation and systolic dysfunction, with a prevalence of 1:2500 in the general population [2]. DCM is one of the leading causes of heart failure and requires cardiac transplantation in severe cases.

Recent advances in genomics have shed more light on the molecular pathogenic mechanisms of cardiomyopathies, contributing to substantial advances in the diagnosis of the disease. Disease-causing variants in many genes are involved in cardiomyopathies, especially those in genes encoding cytoskeletal, sarcomere, and nuclear envelope proteins. Disease-causing variants in more 100 genes encoding proteins involved in many different subcellular systems have been identified to contribute to the genetic landscape of cardiomyopathies, indicating the diversity of pathways contributing to cardiac remodeling [3].

Phospholamban (PLN) is a 52-amino acid integral membrane protein that plays an essential role in regulating cardiac contractility via a reversible inhibitory association with the sarcoplasmic reticulum Ca^2+^ATPase (SERCA2a), the enzyme responsible for maintaining calcium homeostasis in the heart muscle. Pathogenic variants in the *PLN* gene may cause inherited cardiomyopathies due to a key role in the function of the sarcoplasmic reticulum (SR) which is the main dynamic Ca^2+^ storage compartment of the cell [4,5]. The Ca^2+^ is released from the SR into the cytosol, facilitating contraction of the myocytes and is transported back into the SR to initiate relaxation by the SERCA2a, the activity of which is regulated by PLN [6,7]. The *PLN* NM_002667.5, c.116T>G variant creates a premature translational stop signal (p.Leu39*) in the *PLN* gene, which is predicted to lead to a truncated protein, as the last 14 amino acids are lost. This alteration has been previously identified in multiple individuals with DCM [8], HCM [9,10,11,12,13] and unexplained cardiac arrest in the absence of a cardiomyopathy phenotype [14].

## 2. Materials and Methods

Patients with *PLN* p.Leu39* variants were identified at two tertiary referral centers that routinely evaluate patients with HCM/ DCM. Each center independently identified all genotype positive individuals among the consecutive HCM and DCM patients for whom clinical genetic testing had been performed for HCM or DCM. All probands and family members with confirmed *PLN* p.Leu39* variant were included to enable study of the variability in phenotypes of mutation carriers. Retrospective data were collected into a prespecified database.

Patients were given annotations based on their family status: the first digit stands for the family, and the second digit stands for the individual within the family (the proband has 1 as the second digit.

HCM or DCM was diagnosed by physical examination, electrocardiogram, echocardiogram and magnetic resonance imaging, according to the criteria of the ESC working group on cardiomyopathies [15]. Written informed consent was obtained from all patients before enrollment. Next-generation sequencing (NGS) was performed in 189 patients with HCM and 62 patients with DCM phenotype using cardiomyopathy dedicated gene panels: Invitae Dilated Cardiomyopathy and Left Ventricular Noncompaction (80 genes tested), Invitae Hypertrophic Cardiomyopathy Panel (44 genes tested)—INVITAE laboratory, United States, Hypertrophic Cardiomyopathy Panel (92 genes tested)—Blueprint Genetics laboratory, Finland or TruSightCardio Panel Illumina (174 genes tested)—Genomic Center of the University of Medicine and Pharmacy Victor Babeș Timișoara, depending on the patient and physician preferences, considering different turnaround time and reimbursement.

The sequencing was carried out by the sequencing instruments using the Illumina (San Diego, CA) sequencing-by-synthesis method. All sequence reads were mapped onto the human reference genome hg37. The American College of Medical Genetics and Genomics and the Association for Molecular Pathology (ACMG/AMP) was used for classification of genetic variants as benign (B), likely benign (LB), variant of uncertain significance (VUS), likely pathogenic (LP), and pathogenic (P) [16]. Results were categorized as disease causing/significant (if P or LP), negative (if B or LB), or uncertain (VUS), depending on the classification of the variant identified and the inheritance pattern of the associated condition.

Detailed clinical data were collected on all patients, at each tertiary care center, by reviewing the clinical records from the most recent visit of all participants and included pertinent personal (symptom onset and its aggravation) or family history (especially with regard to HCM or SCD with a three-generation family history) and treatment strategies. Biomarkers such as natriuretic peptides and creatine kinases were assessed. Echocardiographic parameters such as left ventricle (LV) ejection fraction (LVEF), LV end diastolic and systolic diameter (LVEDD, LVESD) and volume (LVEDV, LVESV), global longitudinal strain (GLS), wall thickness, LV outflow tract (LVOT) or mid-cavity pressure gradient, left atrium (LA) systolic volume and diastolic function parameters were determined. When available, cardiac magnetic resonance (CMR) findings were described: LVEF, LV end diastolic and systolic volumes, right ventricle ejection fraction (RVEF), maximum wall thickness, presence of late gadolinium enhancement (LGE). Resting electrocardiogram (ECG) and 24 h electrocardiographic Holter monitoring data were also analyzed. ECG changes were defined as described: ‘high QRS voltage’ is defined as sum of S in V1 and R in V5 exceeded 35 mm, ‘low QRS voltage is defined QRS by a amplitude of less than 5 mm in the limb leads and/or less than 10 mm in the precordial leads, ‘repolarization abnormalities’ are defined as presence of ST segment depression ≥1 mm or an inverted T wave opposite to the QRS axis in at least two contiguous leads, and ‘pathologic Q waves’ are defined as any Q wave with a width greater than 40 ms or a depth greater than one-third of the adjacent R wave. Survival status was identified using the Romanian National Health Insurance House to ensure that no deaths were missed.

Multiple clinical and outcome variables were compared between the phenotype-positive *PLN* group and a control group formed by age matching of phenotype-positive carriers of myosin-binding protein C (*MYBPC3*) or myosin heavy chain (*MYH7*) likely pathogenic or pathogenic variant, to define genotype-phenotype associations. Each *PLN* patients was age-matched with one *MYBPC3* or *MYH7* patient for a better description of the phenotypic evolution. Genotype-positive/phenotype-negative individuals were excluded from the analyses.

Statistical analyses were performed using IBM SPSS 26.0 (IBM Corp, Armonk, NY, USA). Univariable analysis was applied to both continuous and categorical variables. Normality was determined by the Shapiro–Wilk test. Continuous variables were reported as the mean ± standard deviation and/or as the median and interquartile range (IQR) when appropriate. Among-group comparisons were made using a non-parametric test (Mann–Whitney U test). Categorical variables were reported as counts and percentages. Among-group comparisons were made using a χ^2^ test. Statistics with a 2-sided *p* value < 0.05 indicating significant differences.

Due to the observational nature of this study, the use of anonymized data was based on the individual patient’s informed consent including acceptance of data utilization for research.

## 3. Results

### 3.1. PLN Cohort

Genetic investigations identified a pathogenic variant (NM_002667.5, c.116T>G or p.Leu39*, heterozygous) in the *PLN* gene in 11 probands (Pt 1.1, Pt 2.1, Pt 3.1, Pt 4.1, Pt 5.1, Pt 6.1, Pt 7.1, Pt 8.1, Pt 9.1, Pt 10.1, Pt 11.1) representing 9/189 (4.8%) patients tested for HCM and 2/62 (3.2%) patients tested for DCM. Five more cases (Pt 1.2, Pt 2.2, Pt 2.3, Pt 4.2, Pt 6.2) have been identified as a result of cascade screening in the affected families, including three asymptomatic carriers (Pt 2.2, Pt 2.3, Pt 6.2).

Multiple cardiomyopathy pathogenic variants were present in three patients from this cohort. One patient (Pt 10.1) carries a pathogenic variants in another HCM-related gene [(*MYBPC3*] NM_000256.3, c.1504C>T, p.Arg502Trp,) while two others (Pt 6.1 and Pt 11.1) carry a likely pathogenic variant ([*MYH7*] NM_000257.4, c.5134C>T, p.Arg1712Trp and titin [*TTN*] NM_001267550.2, c.94128del, p.Tyr31376*, respectively).

The clinical variables of patients enrolled in this study at the last visit are displayed in Table 1. Six patients (Pt 1.1, Pt 1.2, Pt 2.2, Pt 2.3, Pt 4.2 and Pt 6.2) had a family history of HCM, and another four (Pt 3.1, Pt 5.1, Pt 7.1, Pt 9.1) had a family history of SCD. At the initial presentation to our clinic, 4 (36%) out of 13 affected individuals were asymptomatic, but 9 of them had already developed heart failure symptoms (at least NYHA class II), while 5 patients complained of palpitations and 5 patients had angina. Syncope was recorded only for patient 7.1 and patient 11.1. The median age at the diagnosis was 46 years old, with a wide range reported (4 to 66). Pt 6.1 exhibited early-onset cardiomyopathy (during his first years of life) while his father (Pt 6.2, carrying the same PLN variant) is phenotype negative. The presence of an additionally HCM-related variant in the child (MYH7) could potentially explain the severity of the phenotype in this case. A total of 9 patients developed atrial fibrillation (AF), with a median age of onset of 54 (42.5–65) years old.

**Figure 1:**
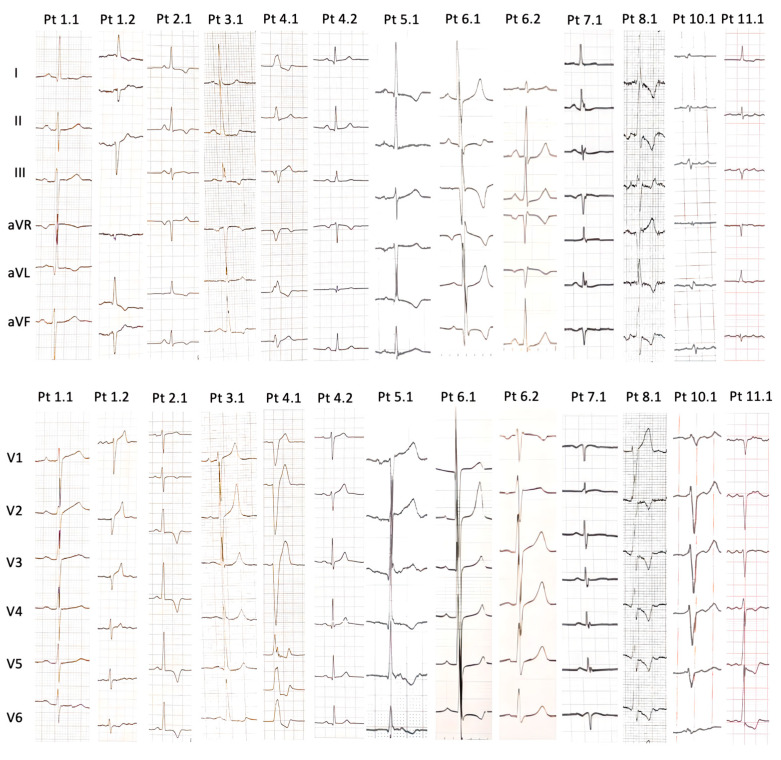
Findings of 12-lead electrocardiograms of PLN patients (when available).

The N-terminal pro B-type natriuretic peptide (NTproBNP) levels were significantly elevated (median value 4893.2 ± 6299.5 pg/dL). At 12-lead ECG examination (Figure 1), six patients (Pt 2.3, Pt 3.1, Pt 4.1, Pt 5.1, Pt 6.1, Pt 8.1) presented with high QRS voltage, and three patients had low QRS voltage (Pt 9.1, Pt 10.1, Pt 11.1). ECG of nine of the patients (Pt 1.1, Pt 1.2, Pt 2.1, Pt 3.1, Pt 4.1, Pt 5.1, Pt 6.1, Pt 6.2, Pt 8.1, Pt 11.1) showed inverted T waves. Pt 4.1, Pt 6.1, Pt 6.2, Pt 10.1 and Pt 11.1 developed pathological Q waves. Additionally, one patient (Pt 4.1) had a left bundle branch block morphology.

Echocardiographic and magnetic resonance imaging features are presented in Table 2. The majority of *PLN* subjects displayed a hypertrophied phenotype (8 individuals representing 57%) mostly with septal thickening, but also with concentric hypertrophy (Pt 1.2, Pt 5.1, Pt 7.1) or apical hypertrophy (Pt 2.1, Pt 11.1), but a dilated phenotype (2 patients) or a mixed one (3 patients) characterized by a dilated and hypertrophied LV were noted. The maximal LV walls thickness ranged from 9 mm to 30 mm (median 17 mm). Right ventricle (RV) involvement was noted in 10 patients.

Five patients (Pt 1.2, Pt 4.1, Pt 9.1, Pt 10.1, Pt 11.1) showed decreased LVEF; coronary artery disease could have been involved in the first case, but was absent for the other four individuals. LVOT dynamic obstruction was present in two cases (Pt 1.1, Pt 3.1). Another patient (Pt 2.1) experienced mid-cavity obstruction.

**Figure 2:**
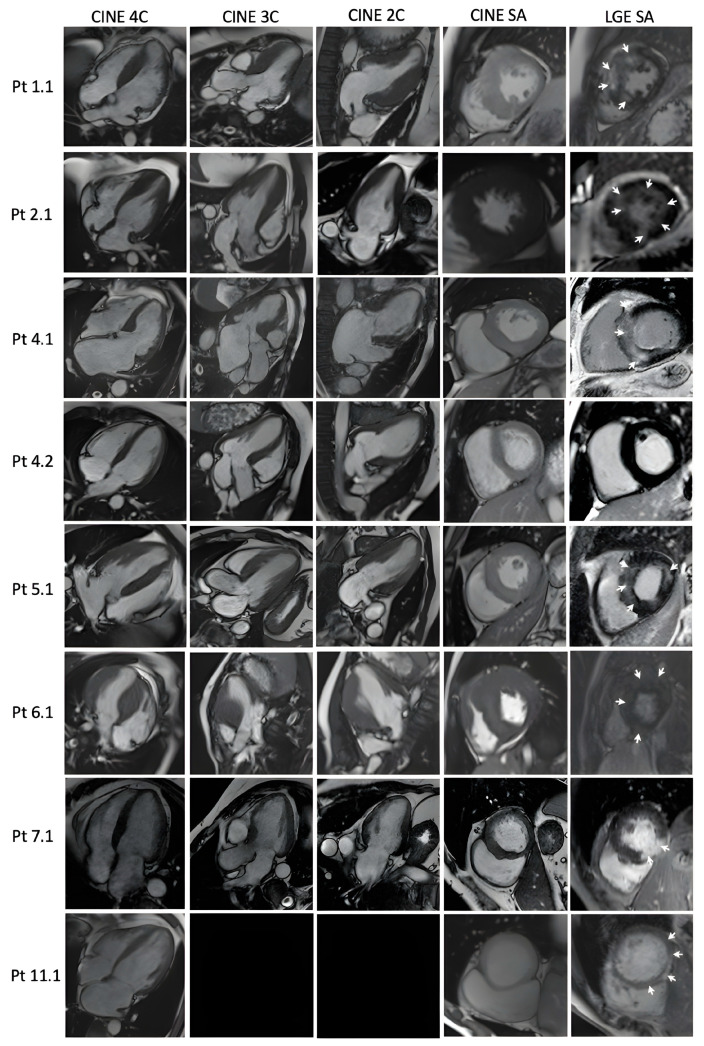
Cardiac magnetic resonance findings in PLN patients: Diastolic frames of cine images in 4, 3, 2 chambers and short axis, and late gadolinium enhancement images in short axis, respectively. Black rectangles: the image is not available. White arrows demonstrate areas of LGE.

Only nine patients were examined with CMR (Figure 2). Therefore, eight (89%) of them exhibited LGE.

Echocardiographic images from *PLN* patients can be seen in Figure S1 from Supplementary Material.

### 3.2. Genotype-Phenotype Correlation and PNL-Other Sarcomeric Gene Comparison

Phenotype-positive *PLN* carriers were included in the *PLN* group and phenotype-positive *MYBPC3/MYH7* carriers matched for age were included in the *MYBPC3/MYH7* group. There was no difference between groups with respect to gender, age at diagnosis, incidence of AF and the frequency of symptoms. Echocardiographic and CMR features did not show any statistical difference between groups, except for left ventricle volumes (LVEDVi and LVESVi), *PLN* patients had larger LVs compared to the *MYBPC3/MYH7* group (*p* = 0.045 and 0.050, respectively). Regarding ECG findings, the *PLN* group showed more frequent pathological Q waves (*p* = 0.030), but conduction anomalies (bundle branch blocks) were rarely seen in these patients (*p* = 0.047). Patients from the *PLN* group experienced more frequent non-sustained VT than the *MYBPC3/MYH7* group (*p* = 0.027). We did not detect significant differences in treatment between the two groups (Table 3).

Patient phenotypes, clinical course and family pedigree charts for each *PLN* p.Leu39* individual are described in the Case Series from Section S1 in the Supplementary Material.

## 4. Discussion

Phospholamban has emerged as a critical regulator of Ca^2+^ homeostasis. Both the inhibition and overexpression of phospholamban have been associated with the development of primary cardiomyopathies in humans [9,17,18]. The human *PLN* gene is located on chromosome 6, and the amino acid sequence of PLN is highly conserved in all species [19].

Worldwide, several *PLN* variants have strong association with variable cardiac phenotypes ranging from dilated cardiomyopathy [8,17] to hypertrophic [9,10,11,12,13,18] and even arrhythmogenic cardiomyopathy (ACM) [20,21], but the causative defects leading to cardiomyopathies remain incompletely elucidated.

Different studies suggest that the p.Leu39* PLN mutant is expressed but mis-located within the cardiomyocytes [22,23]. The mis-location of PLN was associated to decreased SERCA2a expression and impaired Ca^2+^ handling in human pathophysiology. Calcium plays a crucial role in cardiomyocytes by acting as a signal that controls the contraction-relaxation cycle and cardiac hypertrophy. Increased Ca^2+^ levels in myoplasm contribute to the development and progression of hypertrophy [24].

In this study, we summarized the clinical findings of 16 patients carrying the *PLN* p.Leu39* variant in Romania, including 13 phenotype-positive individuals and 3 asymptomatic carriers. The phenotype of p.Leu39* variant carriers in the *PLN* gene has been shown to vary considerably, ranging from the typical HCM phenotype, to a mixed phenotype (dilated and hypertrophied LV) and DCM phenotype. Cardiomyopathy patients often present with highly variable phenotypic expressions in terms of structural and functional parameters of the heart and clinical course. It is common to observe patients from the same family, who share a gene variant, that exhibit variable phenotypes ranging from almost asymptomatic forms to the development of heart failure or to severe arrhythmias/sudden death. As was presented above, the *PLN* group presented incomplete and age-dependent penetrance, the onset of the symptoms having a considerably large range (from 4 to 66 years old) or the individual never developing the disease (as was the case with patient 6.2). While most of the patients had a “benign” evolution of the disease, with no sustained malignant arrhythmias and no family history of SCD, there is also the case of patient 9.1 and her three siblings who suffered youthful disease-related deaths. It is well known that patient-specific factors beyond the single pathogenic variant or environmental modifiers, can dramatically modulate the phenotype in different individuals [25,26].

In agreement with previous findings [12], the majority of our *PLN* p.Leu39* patients demonstrated cardiac hypertrophy, supporting the hypothesis that p.Leu39* is an HCM-predisposing variant. Moreover, two patients (Pt 4.1 and Pt 10.1) were firstly diagnosed with LV hypertrophy in the presence of a normal LV systolic function, but several years later after the diagnosis the LVEF started declining, the echocardiographic aspect could be linked to a progression to end-stage dilated HCM.

Haghighi et al. identified two probands with DCM and *PLN* p.Leu39* variant, diagnosed at 27, respectively 28 years old [9]. Nine individuals from both families of probands underwent echocardiographic examination, revealing that they all had normal LVEF, but four of them exhibited left ventricular hypertrophy. Because of the presence of hypertrophy in some members of both families, we could assume that the dilated phenotype in the probands might have been a consequence of evolution from a hypertrophic phenotype to a burnout stage. However, genome-wide association studies demonstrated that many loci are associated with both HCM and DCM [27]. Despite the genetic overlap, distinct disorders might develop through opposing genetic effects [28] between PLN function and HCM phenotype, and a possible evolution to a burn-out stage.

Abrams et al. reported the existence of a *PLN* p.Leu39* variant in a patient without any imaging evidence of structural heart disease, but with an arrhythmogenic phenotype, developing ventricular tachycardia and ventricular fibrillation storm [29]. It is well known that the *PLN* p.Arg14del founder variant (NM_002667.5) is characterized by life-threatening ventricular arrhythmias and sudden cardiac death [30,31,32], highlighting a potentially primary arrhythmogenic nature of this variant. During the follow-up, we demonstrated that in our *PLN* cohort, non-sustained VT is more frequent than in those with sarcomeric gene variants (*p* = 0.027), even the HCM Risk SCD scores were similar (Table S1) which emphasize the importance of detecting this variant. Three patients (Pt 3.1, Pt 7.1, Pt 9.1) had a positive family history for SCD in first- and second-degree relatives, some of whom died at very young ages (18, 21, 25, 30, 58 years old). Another patient (Pt 5.1) had two sons who died very early in life, but this could be attributed to a potassium voltage-gated channel [*KCNQ1*] c.604G>A variant (NM_000218.3), which was detected in the father in addition to the *PLN* variant.

Low QRS voltage is a common finding in *PLN*-related DCM; however, the study cohorts consisted only in patients with the p.Arg14del variant [30,33,34]. Similarly, in this p.Leu39* cohort, both patients diagnosed with a dilated phenotype (Pt 9.1, Pt 11.1) experienced low voltage electrocardiogram. Additionally, Pt 10.1 diagnosed with HCM with evolution to end-stage disease due to the extensive fibrosis, had a similar ECG pattern. One more patient (Pt 7.1) with an eccentric LV hypertrophy and a maximum wall thickness (MWT) of 17 mm showed disproportion to electrocardiographic QRS voltage. Another ECG abnormality found in the study group is the presence of pathological Q waves, which are substantially more common than in the *MYBPC3/MYH7* group (*p* = 0.030). A distinct fibrosis pattern was reported for the PLN p.Arg14del, with extensive subepicardial fibrosis in the posterolateral wall [35], but this pattern could not be confirmed for our PLN p.Leu39* cohort with a hypertrophic phenotype, where the LGE involved mostly hypertrophied segments, similar to sarcomeric variants, but was present for the dilated phenotype (Pt. 11.1).

Previously published studies reported *PLN* variants as a rare cause of hypertrophic or dilated cardiomyopathies [18,36], with the exception of p.Arg14del variant which was found with higher frequency among patients with DCM and ACM [31,37]. While *PLN*- disease-causing variants count for a small percentage of patients with HCM among different publications [10,12], in this cohort the percentage of *PLN* carriers was relatively high (4.8%), highlighting the importance for routinely screening for *PLN*, alongside historically validated genes for HCM.

Furthermore, this is the first study to analyze the differences in clinical and paraclinical characteristics between patients with *PLN* p.Leu39* and those with the most commonly HCM-associated variants (*MYBPC3/MYH7*). After matching the two groups by age, we observed that the evolution of the *PLN*-related disease was similar to that related to sarcomeric genes variants, but the *PLN* patients are at greater risk for ventricular arrhythmia.

The main limitation of this study is the sample size, which was small. However, this is common in many studies dealing with rare diseases. Despite the fact that genetic results came from different laboratories, and employed gene panels of different size, the methodology is similar and all panels include the core sarcomeric genes associated with cardiomyopathies. To our knowledge, this is the largest cohort of patients carrying this pathogenic variant. Thus, future studies identifying the *PLN* p.Leu39* variant are required to elucidate the potential association.

## 5. Conclusions

Our results demonstrate that *PLN* mutant p.Leu39*-related cardiomyopathy is mainly characterized by a hypertrophic phenotype with a potential to evolve towards a dilated phenotype. We hypothesized that the dilated phenotype could be related to HCM evolution into the end-stage phase, but further studies are required for a better understanding of the observed phenotype in human carriers. In addition, the genotype–phenotype relationship in *PLN* patients is not distinctly different from those caused by other causal genes (*MYBPC3/MYH7*), but these patients could carry a high risk for ventricular arrhythmia. Our findings contribute to a better understanding of the cardiac effects and consequences of the chronic expression of the p.Leu39* mutant in humans.

## Figures and Tables

**Table 1:** Baseline Characteristics of PLN patients.

Characteristic	Pt 1.1	Pt 1.2	Pt 2.1	Pt 2.2	Pt 2.3	Pt 3.1	Pt 4.1	Pt 4.2	Pt 5.1	Pt 6.1	Pt 6.2	Pt 7.1	Pt 8.1	Pt 9.1	Pt 10.1	Pt 11.1
Age	54	59	48	49	17	69	74	42	52	10	50	55	74	37	68	49
Sex	F	M	M	F	M	F	M	F	M	M	M	F	M	F	M	M
Age at disease onset	33	54	47	-	-	54	62	42	51	4	-	54	66	30	57	44
Family history																
HCM	+	+	-	+	+	-	-	+	-	-	+	-	-	-	-	-
SCD	-	-	-	-	-	+	-	-	+	-	-	+	-	+	-	-
Symptoms																
NYHA Class	II	II	II	-	-	II	II	I	I	I	-	II	I	IV	II	III
Syncope	-	-	-	-	-	-	-	-	-	-	-	+	-	-	-	+
Palpitations	+	+	+	-	-	-	-	-	-	-	-	-	-	-	+	+
Chest pain	+	-	+	-	-	+	-	-	-	+	-	+	-	-	-	-
ECG abnormalities																
High QRS voltage	-	-	-	-	+	+	+	-	+	+	-	-	+	-	-	-
Low QRS voltage	-	-	-	-	-	-	-	-	-	-	-	-	-	+	+	+
Repolarization abnormalities	+	+	+	-	-	+	+	-	+	+	+	-	+	-	-	+
Pathologic Q waves	-	-	-	-	-	-	+	-	-	+	+	-	-	-	+	+
Other LP/P cardiomyopathy variants	-	-	-	-	-	-	-	-	-	+	-	-	-	-	+	+

Legend: ECG: electrocardiogram, F: female, HCM: hypertrophic cardiomyopathy, LP: likely pathological, M: male, NYHA: New York Heart Association, P: pathological, SCD: sudden cardiac death, +: this characteristic is present, -: this characteristic is not present, I, II, III, IV: NYHA classes.

**Table 2:** Echocardiography and cardiac magnetic resonance characteristics of PLN patients.

Characteristic	Pt 1.1	Pt 1.2	Pt 2.1	Pt 2.2	Pt 2.3	Pt 3.1	Pt 4.1	Pt 4.2	Pt 5.1	Pt 6.1	Pt 6.2	Pt 7.1	Pt 8.1	Pt 9.1	Pt 10.1	Pt 11.1
Phenotype	hyp	mix	hyp	-	-	hyp	mix	hyp	hyp	hyp	-	hyp	hyp	dil	mix	dil
ETT																
LVEDD/LVESD (mm)	46/26	45/40	41/24	38/23	42/26	44/30	57/29	37/25	41/27	28/15	38/25	44/28	51/29	52	57/51	59/43
LVEDVi/LVESVi (mL/m^2^)	57/19	52/31			50/18		56/37	47/17	76/29		64/24	77/29		57/44	92/72	53/40
IVS/PW (mm)	22/11	15/14	11/10	11/9.5	10/10	18/13	22/12	13/9	16/15	26/8	10/9	16/13	18/11		15/10	9/5
MWT (mm)	30	16	16	11.5	10	20	22	13	16	28	11	17	18		17	9
RVWT (mm)	7	6.5	7	4		9	8	5	7	8		7	8		6	4
LVEF (%)	67	40	70	60	65	70	33	62	62	65	63	62	69	22	21	25
E/A	2.1		1.2	1.1	1.7			1.2	0.8	1	1.5	1.2	0.79	3.8		3.2
E/e’	21	15	9.2	9	7		6	8.5	8		4.5	13	10.9	9		16.3
LVOT/mid-cavity peak gradient ^a^	30	-	48	-	-	90	-	-	-	-	-	-	-	-	-	-
Other ETT findings	AA		AH Cr							AA Cr				tTR	PE	
CMR																
LVEF (%)	70		71				34	54	60	58		61		40		27
RVEF (%)	69		69				38	58	65	53		55				39
LVEDVi (ml/m^2^)	70		70				142	73	84	66		66				121
LVESVi (ml/m^2^)	20		20				94	34	34	27		26				88
MWT (mm)	22		18				20	13	18	23		16				9
LGE	+		+				+	-	+	+		+		+		+

^a^ (rest or provoked) (mmHg). Legend: AA: apical aneurysm, AH: apical hypertropy, CMR: cardiac magnetic resonance, Cr: myocardial crypt, dil: dilated phenotype, hyp: hypertrophic cardiomyopathy, IVS: interventricular septum, mix: mixed phenotype (dilated and hypertrophic), LGE: late gadolinium enhancement, LVEDD: left ventricular end-diastolic dimension, LVEF: left ventricular ejection fraction, LVEDVi: left ventricular end-diastolic volume index, LVESD: end-systolic dimension, LVESVi: left ventricular end-systolic volume index, LVOT: left ventricle outflow tract, MWT: maximal wall thickness, PE: pericardial effusion, PW: posterior wall, RVWT: right ventricle free wall thickness, TTE: transthoracic echocardiography, and tTR: torrential tricuspid regurgitation, +: this characteristic is present, -: this characteristic is not present.

**Table 3:** Overall characteristics of the *PLN* and *MYH7/MYBPC3* patient groups. Values are means ± standard deviation, medians with interquartile ranges and numbers (percentages). *P* values were calculated by the 2-sided unpaired Mann–Whitney U-test for continuous variables, and by chi-square (χ^2^) test for categorical variables. Bold: statistically significant results.

Overall Cohort Characteristics	*PLN* (n = 16)	*MYBPC3/MYH7* (n = 13)	*p* Value
**Baseline characteristics**	data	data	data
Female gender, n (%)	10 (62.5)	7 (53.8)	0.691
Age (years), median (IQR)	51 (43.5–65.7)	55 (43–68)	1.000
Family history of SCD, n (%)	4 (25)	2 (15.3)	0.352
Family history of HCM, n (%)	6 (37.5)	6 (46.1)	0.216
Age at diagnosis (years), median (IQR)	54 (42.5–65)	46.0 (32.5–49.5)	0.342
HCM phenotype, n (%)	8 (50)	9 (69.2)	
DCM phenotype, n (%)	2 (12.5)	0 (0)	
Mixed phenotype, n (%)	3 (18.7)	4 (30.7)	
Apical hypertrophy, n (%)Significant coronary artery disease, n (%)	2 (12.5)	2 (15.3)	1.000
	2 (20.0)	0 (0)	0.119
**Symptoms**			
Dyspnea, n (%)	9 (56.2)	10 (76.9)	0.658
NYHA class ≥ III, n (%)	2 (12.5)	5 (38.4)	0.185
Palpitations, n (%)	5 (31.2)	4 (30.7)	0.680
Syncope, n (%)	2 (12.5)	4 (30.7)	0.352
Angina, n (%)	5 (31.2)	2 (15.3)	0.185
**Biomarkers**			
NTproBNP (pg/mL), mean ± SD	4893.2 ± 6299.5	4815.4 ± 5390.5	0.980
CK (U/l), mean ± SD	75 ± 42	113.6 ± 37.1	0.079
CK-MB (U/l), mean ± SD	28.1 ± 31	26.2 ±7.8	0.082
**ECG findings**			
High QRS voltage, n (%)	6 (37.5)	4 (30.7)	0.680
Low QRS voltage, n (%)	3 (18.7)	2 (15.3)	0.619
Pathological Q waves, n (%)	5 (31.2)	0 (0)	**0.030**
Repolarization abnormalities, n (%)	9 (56.2)	11 84.6()	0.185
LBBB/RBBB morphology, n (%)	1 (6.2)	6 (46.1)	**0.047**
**Holter monitoring**			
Non-sustained VT, n (%)	5 (31.2)	0 (0)	**0.027**
History of Afib, n (%)	9 (56.2)	8 (61.5)	0.680
**Echocardiographic features**			
LVEDD (mm), mean ± SD	45 ± 8.4	46 ± 9.6	0.934
LVESD (mm), median (IQR)	27 (25–30)	27.5 (23.5–39)	0.800
IVS (mm), mean ± SD	15.5 ± 5	17.4 ± 5.4	0.761
PW (mm), mean ± SD	10.6 ± 2.5	11.7 ± 3.1	0.481
MWT (mm), median (IQR)	17 ± 6.1	18.7 ± 4.9	0.930
LVEDVi (mL/m^2^), mean ± SD	61.8 ± 14.1	45.9 ± 19.7	**0.045**
LVESVi (mL/m^2^), mean ± SD	32.7 ± 15.8	21.5 ± 13.9	**0.050**
LVEF (%), median (IQR)	62 (34.7–66.5)	55 (39–69.5)	0.614
LVEF <50%, n (%)	5 (31.2)	4 (30.7)	0.680
Dinamic gradient^b^ > 30 mmHg, n (%)	3 (18.7)	2 (15.3)	0.619
LV GLS (%), mean ± SD	15.1 ± 6.2	14.6 ± 5.8	0.637
E/A ratio, mean ± SD	1.6 ± 0.9	2.7 ± 2.5	0.295
E/e’ ratio, mean ± SD	10.5 ± 4.6	11.2 ± 8.8	0.870
LAVi (mL/m^2^), mean ± SD	49.8 ± 42.6	75.5 ± 29.6	0.320
RVFW thickness (mm), median (IQR)	7 (5.5–8)	6.5 (5–7.5)	0.497
**CMR features**			
LVEDVi (MRI) (mL/m^2^), median (IQR)	71.5 (67–111.7)	72.5	0.889
LVESVi (MRI) (mL/m^2^), median (IQR)	30.5 (21.6–74.7)	24.5	0.296
LVEF (MRI) (%), median (IQR)	59 (37–65.5)	66.5 (59–66.5)	0.230
RVEF (MRI) (%), mean ± SD	55.7 ± 12.2	61.4 ± 6.8	0.370
LV LGE, n (%)	8 (88.8)	6 (100)	0.849
**Scores**			
HCM Risk SCD score (%), mean ± SD	4.3 ± 3.2	4.7 ± 4.7	0.819
**Treatment**			
Medication, n (%)	12 (75)	13 (100)	0.308
ASA/ Septal myectomy, n (%)	1 (6.2)	0 (0)	0.308
ICD, n (%)	5 (31.2)	4 (30.7)	0.680
Pacemaker, n (%)	0 (0)	1 (7.6)	0.308

Legend: Afib: atrial fibrillation, ASA: alcohol septal ablation, BNP: B-type natriuretic peptide, CK: creatine kinase, CK-MB: creatine kinase-MB, CMR: cardiac magnetic resonance, DCM: dilated cardiomyopathy, ECG: electrocardiography, HCM: hypertrophic cardiomyopathy, ICD: implantable cardiodefibrilator, IVS: interventricular septum, LAVi: left atrium volume index, LBBB: left bundle branch block, LVEDD: left ventricle end-diastolic diameter, LVEDVi: left ventricle end-diastolic volume index, LVEF: left ventricle ejection fraction, LV GLS: left ventricle global longitudinal strain, LV LGE: left ventricle late gadolinium enhancement, LVESD: left ventricle end-systolic diameter, LVESVi: left ventricle end-systolic volume index, LVOT: left ventricle outflow tract, MWT: maximal wall thickness, NTproBNP: N-terminal pro B-type natriuretic peptide, PW: posterior wall, RBBB: right bundle branch block, RVEF: right ventricle ejection fraction, RVFW: right ventricle free wall, SCD: sudden cardiac death, and VT: ventricular tachycardia.

## Data Availability

Data are contained within the article and supplementary materials.

## Supplementary Materials

The following supporting information can be downloaded at: https://www.mdpi.com/article/10.3390/jcdd11020041/s1, Figure S1: Echocardiographic findings in *PLN* patients; Section S1: case series; Table S1: Assessment of risk of sudden cardiac death in *PLN* patients with a hypertrophied or mixed phenotype compared to the age-matched *MYBPC3/MYH7* patients.
