# Supplementary material for: Phospholamban p.Leu39* Cardiomyopathy Compared with Other Sarcomeric Cardiomyopathies: Age-Matched Patient Cohorts and Literature Review
Source: J Cardiovasc Dev Dis. 2024 Jan 28;11(2):41. doi: 10.3390/jcdd11020041 (PMC10889724; doi:10.3390/jcdd11020041)
Supplement: Supplementary file 1 [file jcdd-11-00041-s001.zip › jcdd-2830607-supplementary.pdf]

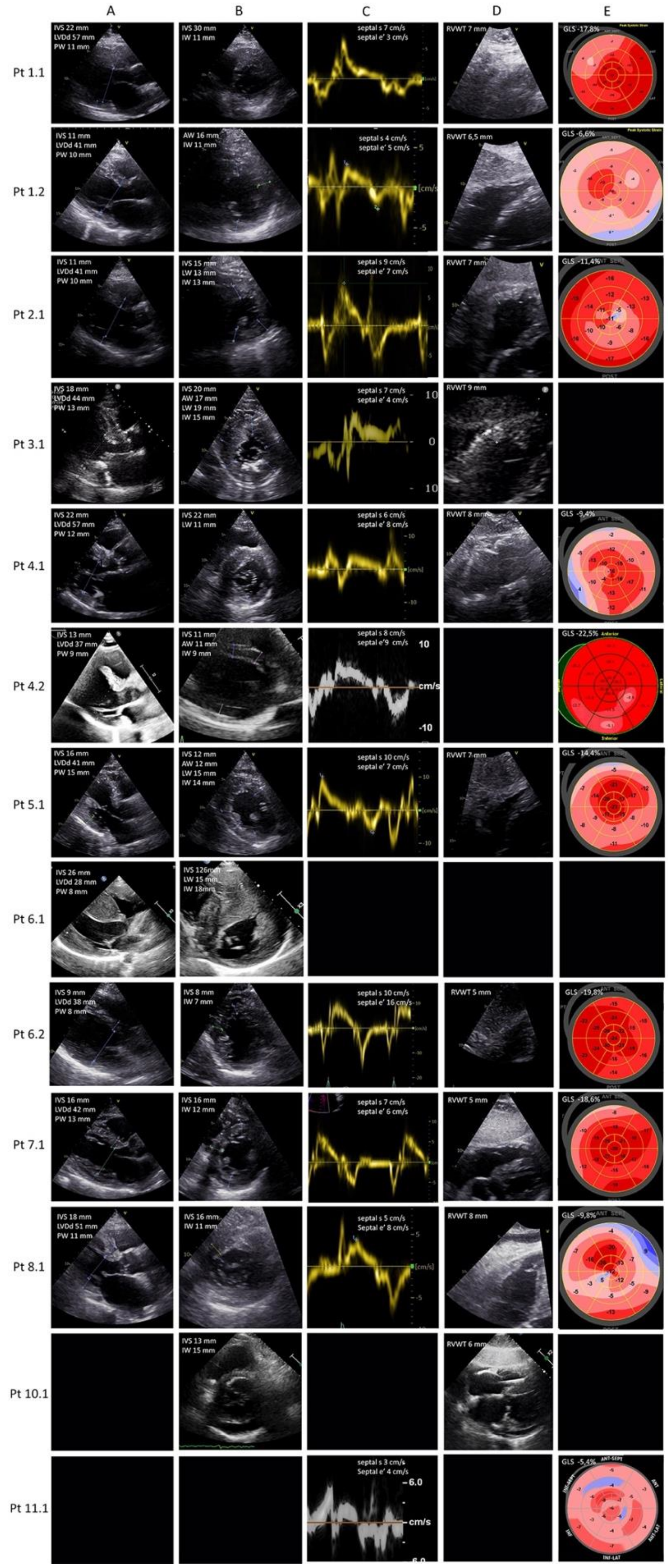
A
B
C
D
E
Pt 1.1
IVS 22 mm
LVDd 57 mm
PW 11 mm
IVS 30 mm
IW 11 mm
septal s 7 cm/s
septal e' 3 cm/s
RVWT 7 mm
GLS -17,8%
Pt 1.2
IVS 11 mm
LVDd 41 mm
PW 10 mm
AW 16 mm
IW 11 mm
septal s 4 cm/s
septal e' 5 cm/s
RVWT 6,5 mm
GLS -6,6%
Pt 2.1
IVS 11 mm
LVDd 41 mm
PW 10 mm
IVS 15 mm
LW 13 mm
IW 13 mm
septal s 9 cm/s
septal e' 7 cm/s
RVWT 7 mm
GLS -11,4%
Pt 3.1
IVS 18 mm
LVDd 44 mm
PW 13 mm
IVS 20 mm
AW 17 mm
LW 19 mm
IW 15 mm
septal s 7 cm/s
septal e' 4 cm/s
RVWT 9 mm
Pt 4.1
IVS 22 mm
LVDd 57 mm
PW 12 mm
IVS 22 mm
LW 11 mm
septal s 6 cm/s
septal e' 8 cm/s
RVWT 8 mm
GLS -9,4%
Pt 4.2
IVS 13 mm
LVDd 37 mm
PW 9 mm
IVS 11 mm
AW 11 mm
IW 9 mm
septal s 8 cm/s
septal e'9 cm/s
GLS -22,5%
Pt 5.1
IVS 16 mm
LVDd 41 mm
PW 15 mm
IVS 12 mm
AW 12 mm
LW 15 mm
IW 14 mm
septal s 10 cm/s
septal e' 7 cm/s
RVWT 7 mm
GLS -14,4%
Pt 6.1
IVS 26 mm
LVDd 28 mm
PW 8 mm
IVS 126mm
LW 15 mm
IW 18mm
Pt 6.2
IVS 9 mm
LVDd 38 mm
PW 8 mm
IVS 8 mm
IW 7 mm
septal s 10 cm/s
septal e' 16 cm/s
RVWT 5 mm
GLS -19,8%
Pt 7.1
IVS 16 mm
LVDd 42 mm
PW 13 mm
IVS 16 mm
IW 12 mm
septal s 7 cm/s
septal e' 6 cm/s
RVWT 5 mm
GLS -18,6%
Pt 8.1
IVS 18 mm
LVDd 51 mm
PW 11 mm
IVS 16 mm
IW 11 mm
septal s 5 cm/s
Septal e' 8 cm/s
RVWT 8 mm
GLS -9,8%
Pt 10.1
IVS 13 mm
IW 15 mm
RVWT 6 mm
Pt 11.1
septal s 3 cm/s
Septal e' 4 cm/s
GLS -5,4%

Figure S1. Ecocardiografic finding in *PLN* patients: A. Parasternal long axis view, B. Parasternal short axis view, C. Tissue Doppler imaging of the septal mitral annular velocities, D. Subcostal view, E. Left ventricular global longitudinal strain. Legend: AW: anterior wall, GLS: global longitudinal strain, IVS: interventricular septum, IW: inferior wall, LVDd left ventricle diastolic diameter, LW: lateral wall, PW: posterior wall, RVWT: right ventricle free wall thickness; Black rectangles: the image is not available.

Section S1. Case series

Patient 1.1 is a 54-year-old woman, previously diagnosed with hypertrophic cardiomyopathy at age 33, experiencing exertional dyspnea (NYHA II class) and angina, sometimes irregular palpitations. She developed atrial fibrillation at the age of 50.

The electrocardiogram revealed lateral T wave inversion, without criteria for diagnosing left ventricular hypertrophy (Sokolow-Lyon 27mm). Transthoracic echocardiogram showed an asymmetrically hypertrophied LV with a small apical aneurysm, with normal LV systolic function with a LVEF of 67% and a LVOT pressure gradient of 30 mmHg at rest and during the performance of a Valsalva maneuver. The CMR confirmed the asymmetric septal and anterior wall hypertrophy with an aneurysmal apex. Transmural LGE was present at the basal and mid interventricular septum (IVS) and anterior wall and at the junction between left and right ventricles. Laboratory results showed high N-terminal prohormone brain natriuretic peptide levels (NTproBNP 2251 pg/mL). She underwent elective coronary angiography showing no evidence of coronary atherosclerosis. Thus, her angina was attributed to the microvascular dysfunction. An exercise-stress echocardiography was performed showing no signs of inducible ischemia or arrhythmias and no increase in outflow tract obstruction. There was a normal blood pressure response, but a reduced exercise tolerance (only 75W). Non-sustained ventricular tachycardia (NSVT) was detected on multiple Holter monitor recordings. Even the HCM risk score for SCD was 5,6%, an implantable cardioverter-defibrillator (ICD) was implanted for primary prevention of sudden cardiac death (indication class IIB, level of evidence C) (15) considering the high arrhythmic risk (repeated episodes of NSVT, apical aneurysm, high LGE extend). She has been receiving treatment with a beta blocker and direct oral anticoagulant. No episodes of ventricular tachycardia (VT) were detected by the ICD yet.

Patient 1.2 is patient's 1.1 brother (Figure S2). He is 59 years old. He developed atrial fibrillation in 2016, when he was also diagnosed with hypokinetic non-dilated cardiomyopathy. He sometimes experiences palpitations and his exercise tolerance is reduced. Because the presence LV global systolic dysfunction, a coronary angiography was performed demonstrating total occlusion of the left circumflex coronary artery (LCx). Percutaneous transluminal coronary angioplasty (PTCA) and stenting were performed for the occluded vessel. Before PTCA, his echocardiography showed a LVEF of 33% with severe hypokinesia of the inferolateral wall. The echocardiography was repeated 3 days after PTCA showing improving of the LVEF (40%), but with the persistence of the regional wall motion abnormality. Biventricular hypertrophy was reported, with a concentric distribution. Meanwhile, his sister was subjected to genetic testing (cardiomyopathy dedicated gene panel) identifying the *PLN c.116T>G* (*p.Leu39**) variant. We then considered that his systolic disfunction is too significant to only be attributed to the single vessel disease, and because the patient also associates biventricular hypertrophy, genetic testing for sister's mutation was recommended. The brother received a positive test result. He was maintained on medical therapy (beta blocker, angiotensin II receptor blocker, direct oral anticoagulant, statin and antiplatelet therapy for 1 year only), the ICD wasn't indicated because of his low HCM risk score for SCD of 1,37%, neither for the reduced LVEF (which is greater than 35%).

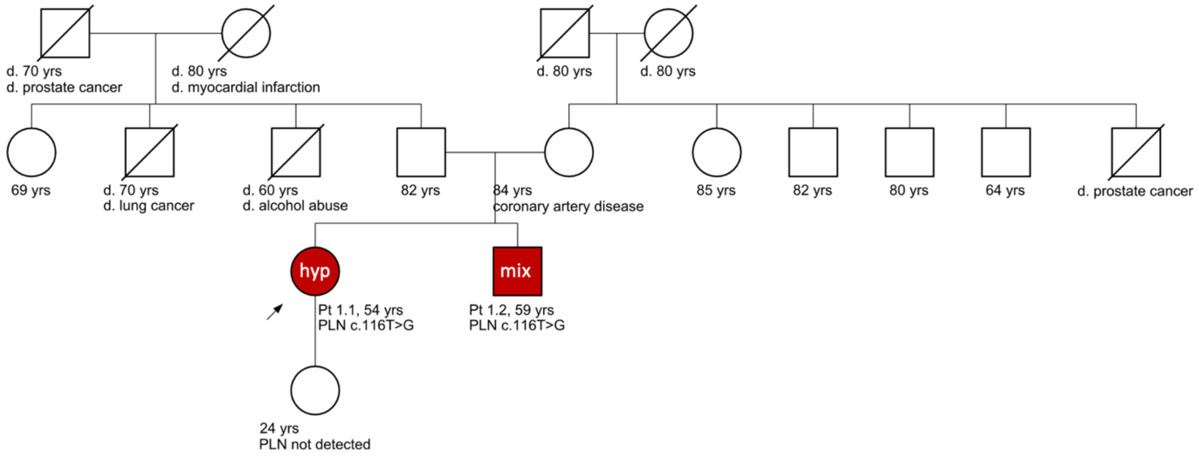

Figure S2. A 4-generation pedigree chart of patient 1.1 and 1.2. Solid black arrow indicates the proband (Pt 1.1); the solid red filled symbol means the presence of *PLN* c.116T>G variant. Legend: hyp: hypertrophic phenotype, mix: mixed phenotype.

Patient 2.1 is a 48-year-old male, recently diagnosed with HCM when inverted T waves were incidentally detected by routinely electrocardiography. His symptoms include exertional dyspnea, angina and intermittent palpitations. Family history of stroke (sister and maternal uncle) was present. One aunt died because of a ruptured cerebral aneurysm. The patient mentioned that his mother might be also diagnosed with HCM (but no documents are available). A paternal uncle required permanent cardiac pacing at advanced age.

Patient 2.2 and **patient 2.3** are patient 2.1's sister and son, respectively (Figure S3). Both of them are asymptomatic carriers, diagnosed through family screening, but the 17 years old son already developed isolated QRS voltage criteria for left ventricle hypertrophy, in the absence of structural changes.

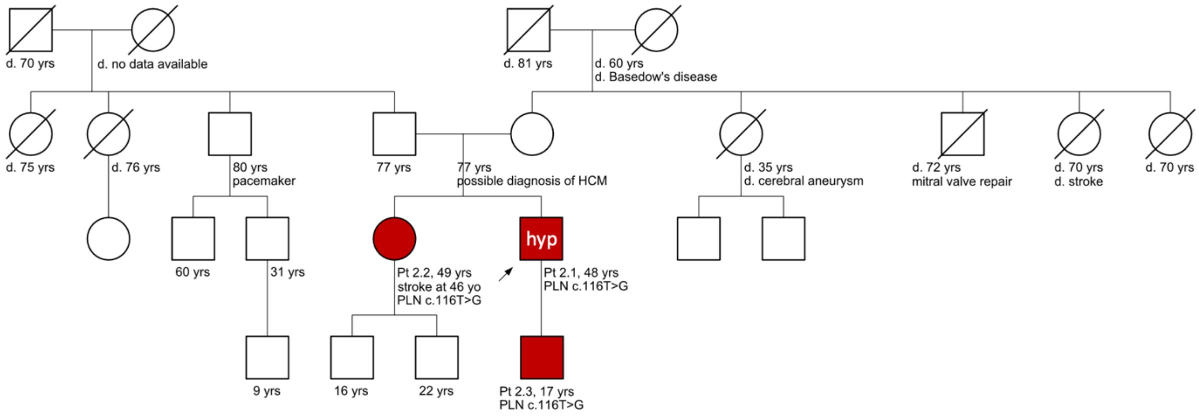

Figure S3. A 4-generation pedigree chart of patient 2.1. Solid black arrow indicates the proband; the solid red filled symbol means the presence of *PLN* c.116T>G variant. Legend: hyp: hypertrophic phenotype.

His ECG showed sinus rhythm, normal QRS voltage with inverted T waves in I, II, aVL, aVF, V3-V6. Holter monitoring identified 2 episodes of NSVT and paroxysmal atrial fibrillation. Echocardiography revealed that the HCM phenotype was apical with mid-cavitary obstruction (48mmHg). The apical segment of the anterior wall had a myocardial crypt. Moreover, CMR study was ordered confirming significant hypertrophy of the apical segments of the LV that was causing obstruction of the ventricular cavity during systole. LGE within the medium septum and apex was described. After the beta-blocker was initiated, the patient remained paucisymptomatic: only mildly reduced functional capacity.

Patient 3.1 is a 69-year-old female, with preexisting cardiac risk factors (hypertension, dyslipidemia, diabetes mellitus) diagnosed in 2010 with hypertrophic obstructive cardiomyopathy (HOCM), evaluated with coronary angiogram (normal coronaries at that time) and a history of a failed alcohol ablation, who was referred to our institution for management of HOCM having dyspnea (NYHA class II) and exertional angina despite optimal medical therapy. Her ECG at presentation showed sinus rhythm with markedly increased LV voltages (Sokolov-Lyon 39mm) with ST segment depression in lateral leads. An echocardiogram on arrival confirmed the known asymmetric hypertrophy of the basal interventricular septum (18 mm), and a worsening of the left ventricular outflow tract obstruction (90 mmHg on provocation) together with SAM dependent moderate mitral regurgitation, mild mitral stenosis and moderate aortic stenosis. There is a family history of SCD (Figure S4) within the maternal line (one aunt died sudden at a very young age). Genetic testing identified the *PLN c.116T>G* variant. The next step in evaluation was to repeat the coronary angiography. This time, coronary lesions were identified: 60-70% left main stenosis, 60-70% LCx stenosis after the origin of the first obtuse marginal branch. After careful consideration by a multidisciplinary group, we decided that the best therapeutical option for the patient will be septal myectomy with concomitant coronary artery bypass grafting (CABG), and eventually surgical aortic valve replacement because of the moderate stenosis and the transaortic approach. The patient agreed with the procedure, signing the informed consent and the surgery was scheduled, but unfortunately, the patient didn't show up at the appointment.

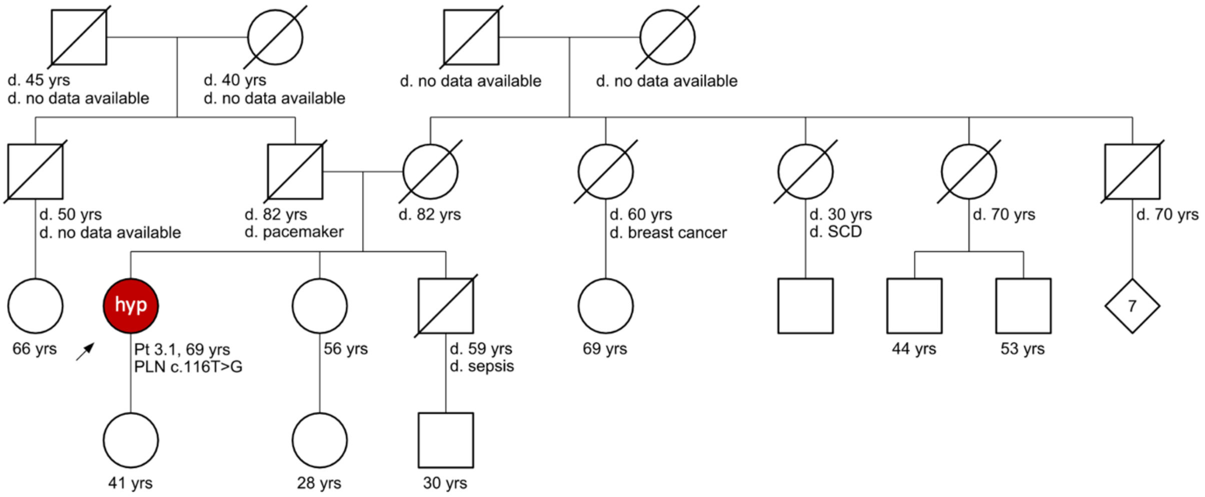

Figure S4. A 4-generation pedigree chart of patient 3.1. Solid black arrow indicates the proband; the solid red filled symbol means the presence of *PLN* c.116T>G variant. Legend: hyp: hypertrophic phenotype.

Patient 4.1 is 74-year-old man, with a history of hypertension and dyslipidemia, known with complete left bundle branch block (LBBB) and permanent atrial fibrillation from age 62. One year ago, he was routine evaluate for the management of hypertension. Echocardiography at that time revealed LV asymmetric septal hypertrophy, moderate reduced left ventricular ejection fraction and a giant left ventricle. There was no outflow tract obstruction at rest or with Valsalva maneuver. Coronary artery disease was excluded by coronary angiography. The resting ECG showed AF and LBBB morphology. CMR evaluation was then realized, describing a dilated LV, with maximum thickness at the septal level of 22 mm and severely reduces ejection fraction (33%). RV dimensions were normal, but the RVEF was also reduced (44%). LGE demonstrates patchy replacement fibrosis at the basal interventricular septum extending at the subendocardial level throw the basal segments of the inferior and infero-lateral walls and at the right ventricular insertion points. After that, he was subjected to genetic testing, with a positive result for *PLN* pathogenic mutation. Cardiac consultation for the patient's daughter and sister was recommended. The patient was treated with beta-blocker, angiotensin receptor II blocker - neprilysin inhibitor, antialdosteronic agent, loop diuretic and direct oral anticoagulant and underwent ICD implantation for the primary prevention of SCD. One year later, the CMR was repeated with similar findings: maximum ventricular septum thickness of 20 mm, LVEF of 34% and RVEF of 38%.

Unfortunately, his daughter inherited the *PLN* mutated gene. She is **patient 4.2** (Figure S5), a young woman of 42 years old, with no previous complaints. She was doing regular daily activities. She denied a history of dyspnea, palpitation, syncope or numbness. Electrocardiographic findings were within normal limits. Holter monitoring showed no cardiac arrhythmias. The echocardiography revealed no significant features except an IVS of 13 mm. Cardiac MRI was done and showed slight hypertrophy of the anterior IVS which measures 13 mm at the basal segment. No LGE was present. The patient is still asymptomatic and no medication was prescribed yet.

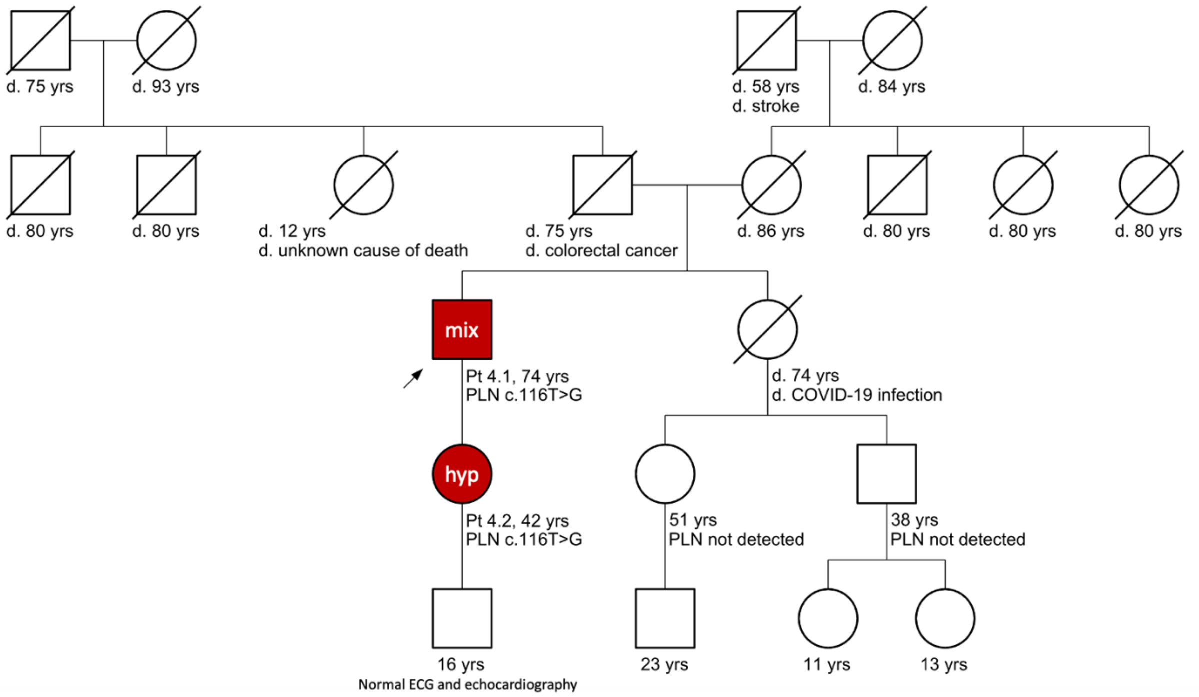

Figure S5. A 5-generation pedigree chart of patient 4.1 and 4.2. Solid black arrow indicates the proband (Pt 4.1); the solid red filled symbol means the presence of PLN c.116T>G variant. Legend: hyp: hypertrophic phenotype, mix: mixed phenotype.

Patient 5.1 is a 52-year-old man with family history of SCD in two sons (Figure S6), with uncontrolled hypertension, who was referred to our institution for evaluation of a possible diagnosis of HCM. Even he has a long history of poorly controlled hypertension (started in the 3rd decade of life), no medication was administered. He is asymptomatic. No history of syncopes. We mention that the younger son died totally unexpected and the other one has a history of multiple "fainting" episodes before he experienced SCD. The ECG showed increased QRS voltages with LV strain pattern. The transthoracic echocardiography demonstrated LV concentric hypertrophy, good systolic function (normal left ventricle ejection fraction), but altered longitudinal dysfunction (low GLS -14,4%), mild mitral regurgitation, without dynamic obstruction of the left ventricle outflow tract. A 24-hours ambulatory ECG monitor showed normal sinus rhythm with isolated ventricular ectopics. Also, ambulatory blood pressure recording was performed for 24-hour showing arterial hypertension: diurnal average BP 224/116mmHg, nocturnal average BP 214/112mmHg (reduced dipping BP pattern), maximum diurnal BP 260/140mmHg. The patient was initatied on anti-hypertensive medications (3 classes) to achieve good blood pressure control. Genetic testing was performed using arrhythmia and cardiomyopathy associate gene panel identifying beside the *PLN* variant another pathogenic variant responsible for long QT syndrome type 1, which could explain the unfortunate death of the 2 sons. The remaining son was also tested for his father's variants and the test was positive only for the *KCNQ1 c.604G>A* (*p.Asp202Asn*). The corrected QT interval of the father was 485 ms. The CMR showed thickness of 18 mm at the basal septum and a circumferential diffuse patchy pattern of

LGE in the LV walls. The risk of sudden cardiac death at 5 years was low, 2,91%. He continued the anti-hypertension medication and a beta-blocker was added. He remains asymptomatic.

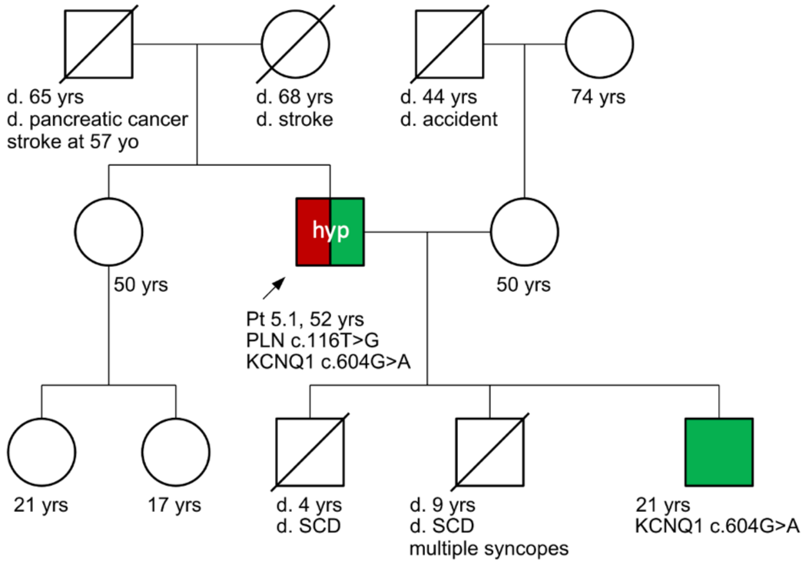

Figure S6. A 3-generation pedigree chart of patient 5.1. Solid black arrow indicates the proband; the solid red filled symbol means the presence of PLN c.116T>G variant; the solid green filled symbol means the presence of *KCNQ1* c.604G>A variant. Legend: hyp: hypertrophic phenotype.

Patient 6.1 is a 10-year-old boy that was diagnosed with HCM very early in life, at 4 years old. From this age, he was under frequent follow-up in the pediatric clinic and a high rate of myocardial thickening was observed. Of note, his family history wasn't significant for a cardiomyopathy. The genetic testing identified two HCM related pathogenic variants: *PLN c.116T>G* (*p.Leu39**) and *MYH7 c.5134C>T* (*p.Arg1712Trp*), the association could contribute to the early onset of the disease and the severe grade of hypertrophy. The resting ECG showed high voltage QRS complexes, Q waves and T waves inversion in inferior leads. Holter monitoring did not demonstrate significant arrhythmias. Clinically, he complained about chest pain. Last echocardiography performed revealed biventricular hypertrophy with maximum ventricular septum thickness of 26 mm (Z score was >20). There was no systolic anterior movement or dynamic LVOT obstruction. A CMR was done for the patient at age 9. Left ventricle was asymmetrical hypertrophied: the medium and basal segment of the IVS measured 23 mm, respectively 21 mm. A crypt was seen in the basal inferior wall and the apex was dyskinetic. The RV was also abnormal, with the hypertrophy of the infundibulum (8 mm). LGE was located in the interventricular septum and at the junction of RV wall into anterior and inferior septum. Furthermore, risk stratification was performed and a recommendation for implantable cardioverter-defibrillator implantation was made because of the high risk of SCD (HCM risk-kids model 10,72% and PRIMACY Childhood HCM SCD Risk Prediction tool 8,78%). Beta-blockade with propranolol was maintained since the diagnosis.

Patient 6.2 is the boy's father, who was screened because of his son's disease. Genetic testing confirmed the presence of the familial mutation in the *PLN* gene; the *MYH7* variant is absent. His ECG had Q waves and T-wave flattening in aVL, with an incomplete right bundle branch block, but no signs of cardiomyopathy at the echocardiography: normal wall thinckness (IVS 10 mm, posterior wall (PW) 9 mm), normal global and longitudinal systolic function (GLS -19,8%), E>A, normal LV filling pressure. He reports no history of any symptoms.

Patient 7.1 is a 56-year-old female, with a family history of 2 SCD (one sister and the son of this sister – Figure S7), who was asymptomatic until 1 year ago when she experienced 3 syncopes. She was also complaining about exertional angina and the coronary arteriography identified multivessel coronary artery disease (60% stenosis of distal right coronary artery, 60% stenosis of mid-distal LCx), but without an indication for revascularization at this moment. A decreased exercise tolerance due to shortness of breath was noted for the past 6 months and the NTproBNP was elevated (751 pg/ml). The echocardiography showed biventricular hypertrophy, even the ECG didn't reveal high LV voltage, but with QRS fragmentation. The ECG Holter monitoring detected 10% of ventricular extrasystoles, without supraventricular or ventricular arrhythmias. The CMR showed transmural fibrosis in the basal and mid segment of the inferior wall (consistent with a possible transmural inferior infarction). The risk for SCD was estimated to be 4,5%.

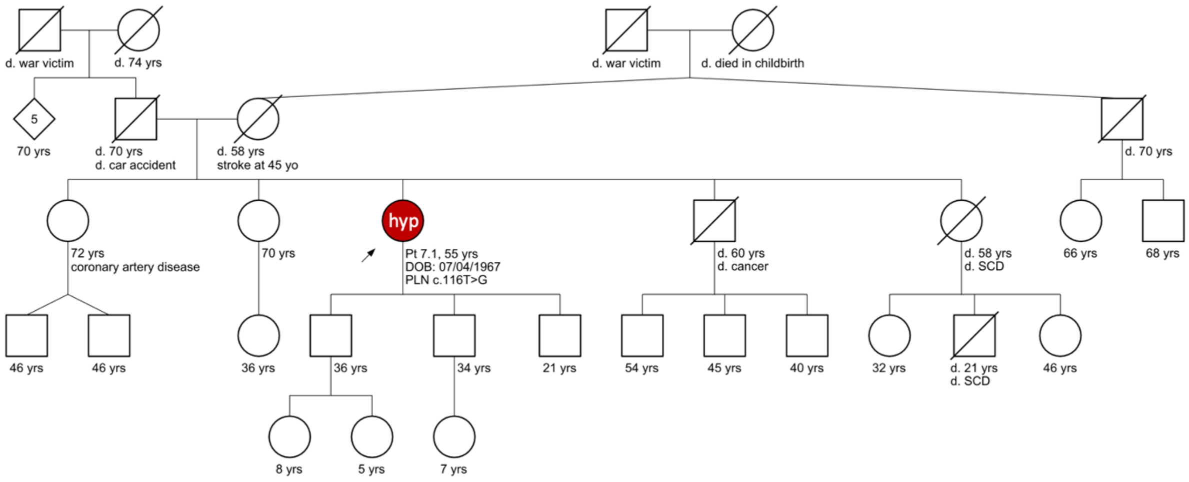

Figure S7. A 5-generation pedigree chart of patient 7.1. Solid black arrow indicates the proband; the solid red filled symbol means the presence of *PLN* c.116T>G variant. Legend: hyp: hypertrophic phenotype.

Patient 8.1 is a 74-year-old asymptomatic man, with no significant family history (Figure S8), incidentally diagnosed with HCM during the surgical work-up prior to renal transplant. A biventricular hypertrophy was noted at the ultrasonography evaluation, with an IVS of 18 mm and RVFW thickness of 8 mm. He also had severe hypertension, requiring high doses of anti-hypertensive drugs to achieve adequate control of

their blood pressure. During 8 years follow-up, his clinical and paraclinical parameters remains stationary. A risk of sudden cardiac death at 5 years of 1,29% indicates there is no need for prophylactic ICD therapy.

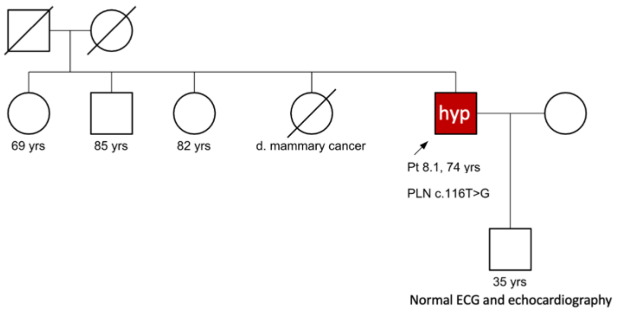

Figure S8. A 3-generation pedigree chart of patient 8.1. Solid black arrow indicates the proband; the solid red filled symbol means the presence of *PLN* c.116T>G variant. Legend: hyp: hypertrophic phenotype.

Patient 9.1 is a young female who died at 36 years old, with multiple hospital admissions for refractory heart failure since she was diagnosed peripartum with DCM with severely reduced ejection fraction (LVEF Simpson biplane 28%) at 30 years old. She had a strong cardiovascular family history: a twin sister with DCM and ICD who died at age 29, a brother with DCM and ICD who died at age 25 and another brother who suddenly died at age 18 (no evaluation prior to his death). Her first hospitalization since the diagnostic, was at 33 years old when she develops NYHA class IV symptoms. At that point, the LVEF was significantly reduced (9%) and she received an ICD for primary prevention of SCD. ICD interrogation documented 27 episodes of non-sustained VT and only one sustained VT episode, terminating spontaneously before therapy. The last echocardiographic examination showed a LVEF of 22% but she continued to deteriorate and to have repeated admissions for acute decompensated heart failure despite maximal optimal therapy. Unfortunately, she finally died because of end stage heart failure.

Patient 10.1 is a 68-year-old man, without an apparent family history, with 4 left atrial ablation procedures for recurrent atrial fibrillation and atrial flutter. At age 57, he was diagnosed with HCM, but 6 years later, the LV was dilated and noncompacted, with severely reduced ejection fraction; hypothesizing about the evolution towards the end stage phase of hypertrophic cardiomyopathy. He associates moderate-sized circumferential pericardial effusion - the effusion measures 16 mm. On the ECG, we remark low QRS voltage in the limb leads with a poor R wave progression in the precordial leads. He still experiences rapid palpitations and dyspnea at exertion. The genetic testing identified another pathogenic mutation (*MYBPC3 c. 1504C>T*) beside the *PLN* variant.

Patient 11.1 is a 49-year-old male, diagnosed with DCM at 44 years old. He has no significant family history. A CMR was performed revealing a moderately dilated LV (LVEDV 253 ml, 121 ml/m2), with severely reduced ejection fraction (27%) and diffuse hypokinesis. LGE in the basal septum (midwall septal stripe) and in

the basal and medium inferior wall (subendocardial) was present. Native T1-values were diffusely increased (1325 ms). The RV was also involved with normal volumes (RVEDV 183 ml, 88 ml/m2) but with an RVEF of 39%. At age 46, he presented sustained VT leading to hemodynamic collapse and he was externally defibrillated. He then received an ICD for primary prevention of SCD. No arrhythmic episodes were detected since then. His ECG shows low QRS voltage in the limb leads, with pseudoinfarction pattern (V1-V3) and inverted T waves in lateral leads. We noted another likely pathogenic variant in the *TTN* gene (c.94128del) with truncation of the TTN protein, which can contribute to the dilated phenotype expressed by the patient.

Table S1. Assessment of risk of sudden cardiac death in *PLN* patients with a hypertrophied or mixed phenotype compared to the age-matched *MYBPC3/MYH7* patients.

Patient	Age	MWT	LA size	LVOT gradient	Family history of SCD	NSVT	Unexplained Syncope	HCM Risk-SCD
Pt 1.1	54	30	48	30	No	Yes	No	5,6%
Pt 1.2	59	16	46	-	No	No	No	1,3%
Pt 2.1	48	16	43	-	No	Yes	No	3,5%
Pt 3.1	69	20	55	90	Yes	Yes	No	9,24%
Pt 4.1	74	22	76	-	No	Yes	No	5,4%
Pt 4.2	42	13	38	-	No	No	No	1,2%
Pt 5.1	52	16	52	-	Yes	No	No	2,9%
Pt 6.1	10	28	28	-	-	No	No	*10,7%
Pt 7.1	55	17	38	28	Yes	No	Yes	4,5%
Pt 8.1	74	18	48	-	No	No	No	1,3%
Pt 10.1	68	17	50	-	No	No	No	1,4%
M 1.1	54	22	56	-	No	No	No	2,6%
M 1.2	55	24	57	-	No	No	No	4,4%
M 2.1	48	16	37	-	No	No	No	1,3%
M 3.1	70	28	49	-	No	No	No	1,7%
M 4.1	68	18	51	-	No	No	Yes	3%
M 4.2	38	14	39	94	No	No	No	2,1%
M 5.1	57	16	47	-	No	No	No	1,4%
M 6.1	11	27	37	-	-	No	No	*17,7%
M 7.1	56	18	61	-	No	No	Yes	4,9%
M 8.1	68	15	46	-	No	No	No	1,1%
M 10.1	68	15	55	-	Yes	No	No	2,2%

*HCM Risk-Kids model was used for Pt 6.1 and M 6.1. Patients were given annotations in the following manner: *PLN* patients are marked as "Pt", while age-matched *MYBPC3/MYH7* patients are labelled as "M".
Legend: HCM: hypertrophic cardiomyopathy, LA: left atrium, LVOT: left ventricle outflow tract, MWT: maximal wall thickness, NSVT: non-sustained ventricular tachycardia, SCD: sudden cardiac death.
